# A high-quality chromosome-level genome assembly of the endangered tree *Kmeria septentrionalis*

**DOI:** 10.1038/s41597-024-03617-1

**Published:** 2024-07-13

**Authors:** Chen-Yu Shi, Guo-Le Qin, Ying-Can Qin, Lin-Yuan Lu, De-Long Guan, Li-Xia Gao

**Affiliations:** Guangxi Key Laboratory of Sericulture Ecology and Applied Intelligent Technology, School of Chemistry and Bioengineering, Hechi University, Hechi, 546300 China

**Keywords:** Conservation genomics, Plant evolution

## Abstract

*Kmeria septentrionalis* is a critically endangered tree endemic to Guangxi, China, and is listed on the International Union for Conservation of Nature’s Red List. The lack of genetic information and high-quality genome data has hindered conservation efforts and studies on this species. In this study, we present a chromosome-level genome assembly of *K. septentrionalis*. The genome was initially assembled to be 2.57 Gb, with a contig N50 of 11.93 Mb. Hi-C guided genome assembly allowed us to anchor 98.83% of the total length of the initial contigs onto 19 pseudochromosomes, resulting in a scaffold N50 of 135.08 Mb. The final chromosome-level genome, spaning 2.54 Gb, achieved a BUSCO completeness of 98.9% and contained 1.67 Gb repetitive elements and 35,927 coding genes. This high-quality genome assembly provides a valuable resource for understanding the genetic basis of conservation-related traits and biological properties of this endangered tree species. Furthermore, it lays a critical foundation for evolutionary studies within the Magnoliaceae family.

## Background & Summary

*Kmeria septentrionalis* Dandy, commonly known as the unisexual magnolia or *Magnolia kwangsiensis* [NCBI Taxonomy ID: 86722], is a remarkable monoecious floral species within the ancient Magnoliaceae family^1,2^. As one of China’s most critically endangered plant species, *K. septentrionalis* holds pivotal significance in maintaining biodiversity^3^. Endemic to China, this species faces numerous challenges to its survival, including limited natural regeneration capacity, a narrow distribution range, and a sparsely distributed population^3,4^. Its primary habitats are confined to the karst limestone mountains of Luocheng and Huanjiang counties in Guangxi province, at elevations ranging from 200 to 750 meters. The Mulun Nature Reserve in Huanjiang County harbors the largest protected population, yet the number of mature individuals remains alarmingly low, below 200^4,5^. This scarcity has drawn urgent attention to the need for conservation efforts. Recognized as a county emblem in Huanjiang and listed as an endangered wild plant at the national level, *K. septentrionalis* necessitates immediate conservation initiatives to prevent further population decline. The International Union for Conservation of Nature’s Red List (IUCNR) classifies *K. septentrionalis* as ‘Critically Endangered’ (CR)^6^. Hence, the pivotal narrative surrounding *K. septentrionalis* in scientific dialogues revolves around species diversity preservation.

Beyond its ecological significance, *K. septentrionalis* provides a unique opportunity to investigate plant evolution. The Magnoliaceae family, considered one of the most ancient extant angiosperm lineages, is a crucial subject in international botanical research aimed at elucidating plant evolutionary history^7,8^. As an outlier within the Magnoliaceae, characterized by distinctive floral sex and propagation mechanisms, *K. septentrionalis* is a key species for understanding evolutionary trajectories within this lineage^1,3,8^. In contrast to the predominantly hermaphroditic flowers observed in most Magnoliaceae members, *K. septentrionalis* exhibits unisexual flowering, representing a unique instance of floral evolution within this ancient family^9–11^. The female flowers of *K. septentrionalis* are considered an evolutionary milestone, having evolved from hermaphroditic ancestors through a process of degeneration.

Moreover, *K. septentrionalis* is the sole known Magnoliaceae member to exhibit apomixis, a unique propagation mechanism that bypasses fertilization to produce seeds. Remarkably, this species possesses the ability to reproduce both sexually and through apomixis, with seeds derived from either method demonstrating germination potential and producing viable seedlings^5,9^. As a dioecious species, *K. septentrionalis* is considered one of the most evolutionarily advanced within its group. Genomic studies of *K. septentrionalis* may provide crucial insights into the complexities of plant evolutionary history, particularly in understanding the evolutionary development of reproductive structures and strategies within the ancient Magnoliaceae family.

The primary objective of this study is to generate a chromosomal-level reference genome for *K. septentrionalis*, which will serve as a vital resource for the conservation of this species’ genetic diversity. As the first to present a high-integrity, high-quality genome assembly of this rare and valuable tree species, we aim to preserve its genetic information and prevent potential extinction. This reference genome will undoubtedly support the continuation of the species and inform future conservation strategies. Additionally, from an evolutionary perspective, the gene annotation information provided herein, particularly the functional gene set, will be invaluable for comparative genomic analyses. This includes investigating the phylogenetic position of *K. septentrionalis*, exploring the origins of unisexual flowers and apomixis, and enhancing our understanding of Magnoliaceae and plant evolution.

## Methods

### Samples and DNA preparation

To prevent any harm to the invaluable trees, we only obtained approximately 30 fresh leaves of *K. septentrionalis* from Huanjiang, Hechi City, in Guangxi Province, China. The collected specimens were then safely stored in nitrogen tanks at Hechi University. Before the collection, approval for leaf gathering was obtained from the local environmental protection agency. About 5 g fresh leaves were employed in the Illumina and PacBio whole-genome sequencing. Hi-C sequencing was performed on another 5 g fresh leaves that apart from the whole-genome sequencing.

For the extraction of high molecular weight (HMW) DNA suitable for PacBio sequencing, we utilized the Qiagen Genomic-tip 100/G Kit (Qiagen, Hilden, Germany), which is designed for the isolation of high-quality, HMW DNA from plant samples. A combination of five leaf samples was processed according to the manufacturer’s protocol, with modifications to optimize the retrieval of long DNA fragments. These modifications included gentle homogenization, extended incubation times, and the use of wide-bore pipette tips to minimize DNA shearing. To ensure the quality and size suitability for PacBio sequencing, we performed size selection using the BluePippin System (Sage Science, Beverly, MA, USA) with a 20 kb cutoff, which allows for the isolation of DNA fragments larger than 20 kb. The selected DNA fragments were quantified and their quality was assessed using the Agilent 2100 Bioanalyzer (Agilent, Waldbronn, Germany) and the Qubit 3.0 Fluorometer (Invitrogen, Carlsbad, CA, USA) to confirm the size, integrity, and quality. Additionally, the integrity of the DNA molecules was evaluated by pulsed-field gel electrophoresis (PFGE) to confirm the presence of high-integrity DNA molecules suitable for PacBio sequencing. For the extraction of regular DNA used in Illumina sequencing, we employed the Qiagen Plant DNA Extraction Mini Kit (Qiagen, Hilden, Germany) according to the manufacturer’s protocol.

### Sequencing and data filtering

For whole-genome sequencing, approximately 5 µg of qualified, high-molecular-weight DNA was used for PacBio library preparation. The SMRTbell Template Prep Kit 1.0 (Pacific Biosciences, Menlo Park, CA, USA) was utilized following the manufacturer’s protocol to prepare the SMRTbell library. The prepared library then underwent the SMRT sequencing process using the PacBio Sequel System (Pacific Biosciences, Menlo Park, CA, USA) in agreement with the manufacturer’s guidelines for long-read sequencing.

For the short-read sequencing, an additional 1 µg of the same high-quality DNA was used to construct a paired-end library using the TruSeq DNA PCR-Free Library Preparation Kit (Illumina, San Diego, CA, USA). This DNA quantity is in line with the recommended input for PCR-free library preparation, ensuring optimal library quality and sequencing performance. Similarly, a separate aliquot of approximately 1 µg of genomic DNA was subjected to chromatin conformation capture (Hi-C) to construct a Hi-C library, aiming to elucidate the three-dimensional genome organization. The Hi-C library preparation involved genome fragmentation with the restriction enzyme MboI and proximity ligation to generate DNA fragments indicative of the chromosomal architecture, following the protocol detailed in the Dovetail Genomics Hi-C Kit (Dovetail Genomics, Scotts Valley, CA, USA). The DNA input quantity for Hi-C library preparation was chosen based on the kit manufacturer’s recommendations to ensure optimal library quality and subsequent sequencing results.

All short-read sequencing libraries, including the paired-end and Hi-C libraries, were prepared with an average insert size of 350 bp. Library quality and concentration were assessed using the Agilent 2100 Bioanalyzer (Agilent, Waldbronn, Germany) and the Qubit 3.0 Fluorometer (Invitrogen, Carlsbad, CA, USA). The prepared libraries were then sequenced on the Illumina NovaSeq 6000 System (Illumina, San Diego, CA, USA) to obtain high-throughput next-generation sequencing data. Sequencing was performed using a paired-end protocol (2 × 150 bp), with image analysis, base calling, and quality score calibration completed by the integrated NovaSeq Control Software (NCS) and Real-Time Analysis (RTA) component.

The final dataset utilized for subsequent analyses in this study comprised Illumina, PacBio HIFI, and HIC data, with a total volume of 138.60 Gb, 473.45 Gb, and 140.81 Gb, respectively. The detailed statistics for these data were provided in Supplement Table S1.

### Genome survey

**Fig. 1:**
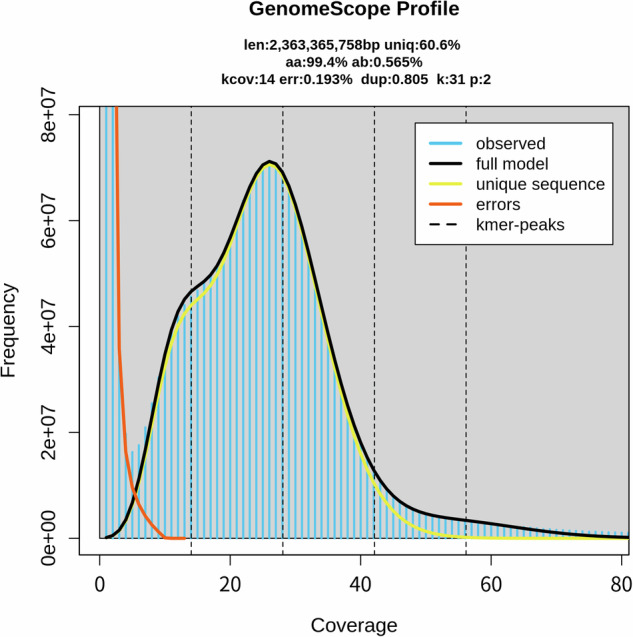
Genome survey based on the Kmer distribution analysis using a Kmer size of 31. The horizontal axis represents the Kmer depth, while the vertical axis represents the corresponding number of Kmers at each depth.

For the comprehensive genome survey, all Illumina clean reads were utilized. The Trimmomatic package v0.38^12^ was employed for quality control of the raw Illumina data. Trimmomatic was employed with the following main parameters: “ILLUMINACLIP: TruSeq. 3-SE.fa:2:30:10 LEADING:3 TRAILING:3 SLIDINGWINDOW:4:15 MINLEN:36”. These parameters were used to clip the TruSeq. 3 adapter sequences, remove low-quality bases, and trim trailing bases where the quality drops below a threshold. The Q30 scores after the quality control ranged from 90.12% to 93.92% for the genome survey sequencing data (Supplement Table S1). Genome characterization was achieved through Kmer analysis. The distribution of 31-mers was calculated using Kmergenie^13^ and GenomeScope v2.0^14^. The ratio of peak values between heterozygous Kmers and homozygous Kmers was computed to assess heterozygosity. The statistics of genome characteristics are presented in Fig. 1, with the x-axis representing Kmer depth and the y-axis representing the corresponding number of Kmers. The genome size was calculated using the formula Kmer-number/depth and was estimated to be approximately 2,363.36 Mb. Additionally, our analysis revealed a heterozygosity rate of 0.565%, and approximately 39.4% of the genome consists of repetitive sequences.

### Genome de novo assembly and evaluation

High-quality long reads generated by the PacBio Sequel system underwent genome assembly using Hifiasm v0.19.4-r575^15^ with parameters of “purge level: -l 2; remove tip unitigs composed of < =INT reads: -n 5”. As a further step, the Purge_dups v1.2.6^16^ was employed to remove the more diverse regions within the assembly with default parameters. Sequencing data were aligned to the preliminary assembly using Minimap2 v2.26^17^ software to refine the assembly with the “PacBio HiFi reads vs reference mapping: map-hifi” parameter. Guided by a genome survey, we conducted genome assembly using HIFI reads. The resulting assembly consisted of 705 contigs, with the longest contig spanning 218.38 Mb and the shortest being 11,778 bp. The total size of the genome scaffolds was 2.57 Gb, slightly smaller than the survey estimation. The GC content of the assembled genome was 40.68%. Assessing the assembly quality, we found that the contig N50 value was 11.91 Mb with an L50 of 63, and the contig N90 was 2.73 Mb with an L90 of 235 (Table 1).

**Table 1:** Statistics for the genome assembly of *K. septentrionalis*.

**Genome assembly**	
the genome scaffolds number	228
the genome contigs number	705
No. of pseudochromosomes	19
the longest length (bp)	218,386,511
the shortest (bp)	11,778
Genome size (bp)	2,572,324,301
the rate of GC (%)	40.68
the scaffold N50 (bp)	135,080,105
the scaffold L50	8
the scaffold N90 (bp)	99,976,078
the scaffold L90	17
the contig N50 (bp)	11,901,512
the contig L50	63
the contig N90 (bp)	2,737,391
the contig L90	235
Size Anchor rate (%)	98.94
BUSCOs (%)	98.9
**Genome annotation**	
No. of protein-coding genes	35,927
Average transcript length (bp)	11,269
Percentage of repetitive sequences (%)	64.87

**Fig. 2:**
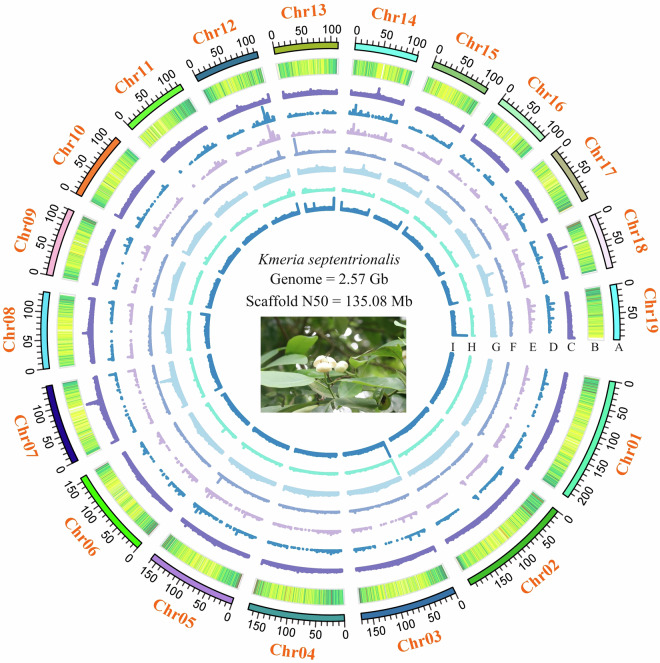
The genomic features of *K. septentrionalis*. (A) The 19 pseudochromosomes, with lengths proportional to their actual sizes; (B) gene density; (C–H) the density of total repeat sequences, LINEs, a combine of LINEs and SINEs, DNA transponsons, LTR and Unknown types; (I) histogram of GC content. The genome size is 2.57 Gb, with a scaffold N50 of 135.08 Mb, indicating high assembly quality. Inset: *K. septentrionalis* flowers in their natural habitat. (C–I) were drawn in 500 kb overlapping sliding windows.

Subsequently, we anchored these contigs into pseudochromosomes utilizing HiC data. The Q30 scores after quality control ranged from 92.29% to 93.61% for the short-read HiC sequencing data (Supplementary Table S1). Before extracting HiC contacts using Juicer v1.10.1^18^, duplicate sequences within HiC reads were eliminated using the MarkDuplicates module from Picard Tools after the initial mapping of sequencing reads to the reference genome. The 3D-DNA v180922^19^ pipeline was employed to achieve a chromosome-level genome assembly. The final high-quality chromosome-level genome assembly was obtained after addressing scaffolding errors by manually adjusting the chromosome boundaries using Juicebox v2.3.0 (map q threshold >30)^20^. The results demonstrated the successful assembly of 496 out of 705 contigs into 19 pseudochromosomes (Fig. 2). The HI-C interaction data confirmed the significance of each scaffold and indicated the accuracy of the scaffolding process (Figure S1). The pseudochromosome lengths ranged from 218.38 Mb to 96.93 Mb. The total size of the genome pseudochromosomes was verified as 2.54 Gb, representing a high anchoring ratio of 98.94%. The GC content was consistently retained at 40.52%, identical to the raw assembly. Upon completion of the pseudochromosome assembly, the scaffold N50 was confirmed as 135.08 Mb, scaffold L50 as 8, scaffold N90 as 99.97 Mb, and scaffold L90 as 17 (Table 1). The 19 pseudochromosomes were ordered based on their length, revealing substantial variation in size. The shortest pseudochromosome, Chr19, spans 96.93 Mb, while the longest, Chr01, extends to 218.38 Mb. The three largest pseudochromosomes, Chr01 to Chr03, each exceed 170 Mbp in length, while the three smallest pseudochromosomes, Chr17 to Chr19, are less than 100 Mbp long. The majority of the pseudochromosomes have lengths greater than 100 Mbp, with 16 out of 19 pseudochromosomes surpassing this threshold (Supplementary Table S2).

The genome assembly was evaluated using Benchmarking Universal Single-Copy Orthologs (BUSCO v5.5.0) analysis against the embryophyta lineage dataset containing 1,614 BUSCO groups. A total of 1,596 (98.9%) BUSCO groups were classified as complete, including 1,531 (94.9%) complete and single-copy BUSCOs and 65 (4.0%) complete and duplicated BUSCOs. Only 8 (0.5%) BUSCO groups were identified as fragmented, and 10 (0.6%) were found to be missing from the genome assembly. These results indicate that the genome assembly captures near-complete gene content, with over 98% of the core embryophyta genes present in full-length forms (Table 1). The low proportion of duplicated and missing BUSCOs further substantiates the high continuity and completeness of the assembled genome. The genome assembly has been deposited at the NCBI GenBank database under the accession GCA_037074715.1, and the annotation GFF3 file is now publicly available with the Zenodo 10.5281/zenodo.10259480.

### Genome annotation

The genome annotation process was divided into three parts: identification of repetitive elements, prediction of non-coding RNAs (ncRNAs), and protein-coding genes (PCGs). We used BLAST v2.14.1^21^ to identify ncRNAs and obtain their sequences and functional annotations by aligning the assembled genome against known ncRNA databases. To identify repetitive elements, RepeatModeler v2.0.2^22^ was employed to construct a novel database, which integrates LTRharvest^23^ and LTR_retriever^24^ to discover complete long terminal repeats (LTRs). Subsequently, the genome assembly was soft-masked for repeats by running RepeatMasker v4.1.2p1^25^ and RMBlast v2.11.0 against the custom repeat library compiled from Repeatmodeler, Dfam 3.5^26^ and Repbase v20181026^27^. The parameters used for RepeatModeler were as follows: -min_score 100 -max_score 1500 -LTR_structure -engine ncbi -species database. Additionally, the Proteinmask analysis was conducted by aligning the genomic sequences against the transposable element protein library provided in RepeatProteinMasker, a package within Repeatmasker enabling the prediction of repetitive sequences. Eventually, a customized script was developed to combine the outcomes obtained from the methods above and eliminate overlapping regions, resulting in a non-redundant dataset. Subsequently, all these repetitive elements were merged into a dataset and then soft-masked from the genome.

Following these steps, we identified approximately 1.67 Gb of repetitive sequences from this genome, constituting about 64.87% of the total size (Table 2). This proportion is slightly lower than the results of previous surveys, a discrepancy potentially attributable to the relatively smaller size of our assembly and the lower sensitivity of current software to short interspersed nuclear elements (SINEs). A more specific breakdown revealed varying relative abundance levels among the different categories of repetitive sequences found in the assembled genome. DNA transposons comprised around 0.54% of the genome, with a total length of 13,834,078 bp. Long interspersed nuclear elements (LINEs) accounted for approximately 0.16% of the genome, adding to a total length of 4,163,525 bp. SINEs appeared almost non-existent, representing a mere 0.00004% of the genome with a total length of only 1,079 base pairs. Interestingly, long terminal repeat retrotransposons (LTRs) accounted for the lion’s share of the genome, taking up approximately 63.29% with a staggering total length of 1.63 Gb. Furthermore, elements of unknown classification made up about 1.45% of the genome, contributing to a total length of 37.20 Mb (Fig. 2, Table 2). Notably, this high proportion of repetitive sequences appears to have a dominant role in the structure of the *K. septentrionalis* genome.

**Table 2:** Summary of Transposable Element (TE) Distribution and Composition in the Genome.

Type	Denovo + Repbase		TE Proteins		Combined TEs	
Size	Length(bp)	% in Genome	Length(bp)	% in Genome	Length(bp)	% in Genome
DNA	13,632,052	0.53	324,702	0.01	13,834,078	0.54
LINE	4,091,062	0.16	101,301	0	4,163,525	0.16
SINE	1,079	0	0	0	1,079	0
LTR	1,573,931,925	61.19	341,239,215	13.27	1,627,981,701	63.29
Unknown	37,200,850	1.45	0	0	37,200,850	1.45
Total	1,618,783,796	62.93	341,665,185	13.28	1,668,715,454	64.87

Note: DNA refers to DNA transposons, which are non-LTR retrotransposons. LINE: Long Interspersed Nuclear Elements (LINEs) are a type of LTR retrotransposon. SINE: Short Interspersed Nuclear Elements (SINEs) are non-LTR retrotransposons. LTR: Long Terminal Repeat (LTR) retrotransposons are a major class of transposable elements. Unknown: This category includes elements that could not be classified into the above-mentioned TE types.

Gene annotation, also known as gene finding, was conducted using a soft-masked genome to distinguish it from subsequent functional annotation. The gene identification process involved a combination of transcript evidence, protein homology predictions, and model-based predictions. The PASA v2.5.2^28^ software was employed to align the transcriptome data to the reference genome and convert it into transcript evidence. For the prediction of homologous protein gene models, the Gemoma v1.6.4^29^ software was utilized. Homologous proteins from the genome of a closely related species, *Magnolia sinica* (GCF_029962835.1) was used for annotation. The Augustus v3.5.0^30^, GlimmerHMM v3.0.1^31^, SNAP v1.0^32^, Geneid v1.4.5^33^, and Genscan v1.0^34^ software were employed to predict gene structure models. Subsequently, all the generated models were integrated using the EVM v1.1.1^35^ software, weighing 10 for transcript evidence, 5 for homologous protein evidence, and 1 for model-based predictions. The merged results were saved in the GFF3 file format for subsequent analysis, including extracting high-quality gene sequences and other analyses.

Our final gene set comprised 35,927 genes, and the integrity of the gene finding results was evaluated using BUSCO v5.5.0^36^ with 98.9% core embryophyte genes found. Among these genes, 94.9% were complete and single-copy BUSCOs, with only 10 missing BUSCOs (0.5%). An analysis of the average length of various gene components showed an average transcript length of 11,269.80 bp, an average coding sequence (CDS) length of 1,092.45 bp, an average of 4.57 exons per gene, an average exon length of 239.14 bp, and an average intron length of 2,852.28 bp. Basic statistics of gene structure prediction, including the number and percentage of annotations from various software, average gene length, and average intron length, are provided in Table 3. Among these genes, 34,800 were distributed on the 19 pseudochromosomes. The number of genes per pseudochromosome varies significantly, ranging from 1,350 on Chr15 to 3,052 on Chr01. The three pseudochromosomes with the highest number of genes are Chr01 (3,052), Chr04 (2,689), and Chr02 (2,724), all of which also rank among the largest in terms of length. Conversely, the pseudochromosomes with the lowest number of genes are Chr15 (1,350), Chr17 (1,353), and Chr07 (1,517) (Supplement Table S2).

**Table 3:** Summary of Gene Prediction Results Using Various Computational Tools.

	Gene set	Number	Average transcript length(bp)	Average CDS length(bp)	Average exons per gene	Average exon length(bp)	Average intron length(bp)
De novo	Augustus	59,723	7,170.54	875.11	3.32	263.25	2,708.61
	GlimmerHMM	130,778	12,959.24	430.77	2.82	152.56	6,870.32
	SNAP	49,355	20,451.77	459.39	3.11	147.87	9,490.19
	Geneid	224,681	3,855.27	466.33	3.02	154.45	1,678.24
	Genscan	120,552	13,958.54	880.34	5.59	157.4	2,847.37
Homolog	Mbi	66,216	4,093.11	619.36	2.67	232.29	2,084.69
	Mgr	45,253	4,754.28	770.17	2.98	258.7	2,015.19
	Lch	46,959	5,717.20	779.77	3.17	245.66	2,270.94
RNAseq	PASA	38,452	11,341.26	1,036.90	4.58	226.19	2,875.00
	Transcripts	53,285	23,740.89	2,319.00	6.55	353.88	3,857.67
EVM		65,265	7,766.02	832.66	3.45	241.14	2,826.45
Pasa-update*		65,093	7,854.09	833.31	3.43	242.8	2,886.80
Final set*		35,927	11,269.80	1,092.45	4.57	239.14	2,852.28

Note: Each gene set is the result of a specific tool’s analysis, and the table provides detailed metrics for each set, including the number of predicted genes, average transcript and coding sequence (CDS) lengths, the average number of exons per gene, and the average lengths of exons and introns.

**Fig. 3:**
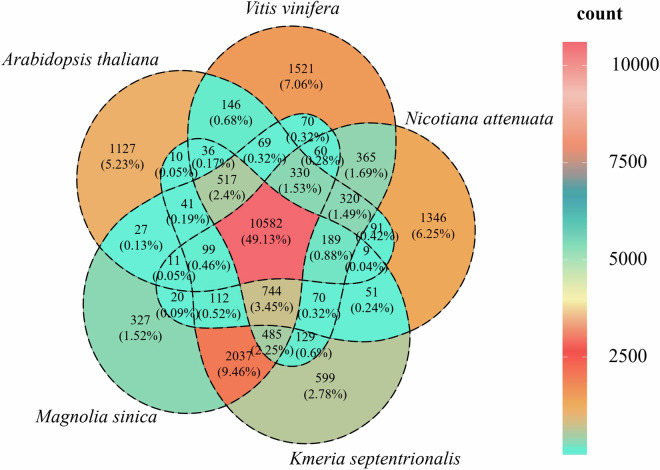
Validation of the accuracy of the assembly and annotation of *K. septentrionalis*. Venn diagram showing the shared and unique gene families among genomes of *K. septentrionalis*, *M. sinica*, *A. thaliana*, *V. vinifera*, and *N. attenuata*. The number and the percentage of gene families in each species is shown in parentheses.

To validate the accuracy of the assembly and annotation, orthologous gene families were inferred and compared with closely related species. OrthoFinder v2.5.5^37^, with Diamond v2.1.8^38^ as the sequence aligner, was used to analyze protein sequences from the *K. septentrionalis* genome and well-assembled genomes of four other plants (*M. sinica*, *Arabidopsis thaliana*, *Vitis vinifera*, and *Nymphaea colorata*). A total of 159,855 proteins from these selected genomes were allocated to 21,560 gene family clusters, with a high proportion of 10,582 clusters (49.13%) being shared among all five genomes. The high consistency in annotation between *K. septentrionalis* and *M. sinica* was further confirmed, with the two species sharing 2,042 unique gene family clusters. The number of clusters unique to *K. septentrionalis* was very low, with only 599 clusters (2.78%)(Fig. 3).

## Data Records

The chromosomal-level genome assembly file was deposited in the NCBI GenBank with accession number GCA_037074715.1^39^. The raw sequencing data for HiFi, Hi-C, and RNA-seq were submitted to the NCBI Sequence Read Archive https://identifiers.org/ncbi/insdc.sra: SRR27346360 - SRR27346366 (DNA sequencing data)^40^, and SRR27866170 - SRR27866178 (RNA sequencing data)^40^. Moreover, the genome annotation files had been submitted at the Zenodo database^41^.

## Technical Validation

The integrity of the isolated DNA was confirmed through agarose gel electrophoresis, while the DNA concentration was determined using a NanoDrop spectrophotometer (NanoDrop products, Wilmington, DE, USA) and a Qubit 3.0 Fluorometer (Life Technologies Corporation, Eugene, OR, USA), with an observed absorbance ratio of approximately 1.80 at 260/280 nm, indicating high purity. The scaffold N50, a measure of the continuity of genome assemblies, achieved a notable enhancement, reaching 135.08 Mb, the highest reported for this species. The impressive 98.9% completeness estimated by Benchmarking Universal Single-Copy Orthologs (BUSCO) underscores the high quality of the assembled genome. Furthermore, the low percentage (0.4%) of the chromosome-level genome involving duplicated single-copy genes, as assessed by BUSCO, suggests that duplication did not significantly impact the genome assembly.

To assess the comprehensiveness of the genome assembly, we employed the sequence identity methodology, whereby HiFi reads were strategically chosen and re-aligned to the assembled genome using Minimap2 v2.26-r1175 software. The coverage per scaffold ranged from 77.76% to 88.92%. Additionally, we utilized the CRAQ v1.0.9 software to dissect and quantify various genomic regions using all short reads. The mapping rate was remarkably high at 99.73%, suggesting that nearly the complete genomic landscape was accurately captured. The coverage rate was also notably high at approximately 99.89%, indicating a high confidence level in our genomic assembly. Importantly, the Adjusted Quality Index was recorded at 91.02, which confirmed the overall quality index according to the software’s instructions. The cumulative evidence collected testifies to the successful acquisition of a high-quality genome assembly for *K. septentrionalis*.

### Supplementary information

Figure S1

Supplement Table S1

Supplement Table S2

## Data Availability

The study utilized freely available software to the public, and the parameters are explicitly outlined in the Methods section. All commands and pipelines were executed following the manuals and protocols of the corresponding bioinformatic software. The study did not utilize custom scripts or code.
