# Supplementary figures and images for: A high-quality chromosome-level genome assembly of the endangered tree Kmeria septentrionalis
Source: Sci Data. 2024 Jul 13;11:775. doi: 10.1038/s41597-024-03617-1 (PMC11246460; doi:10.1038/s41597-024-03617-1)

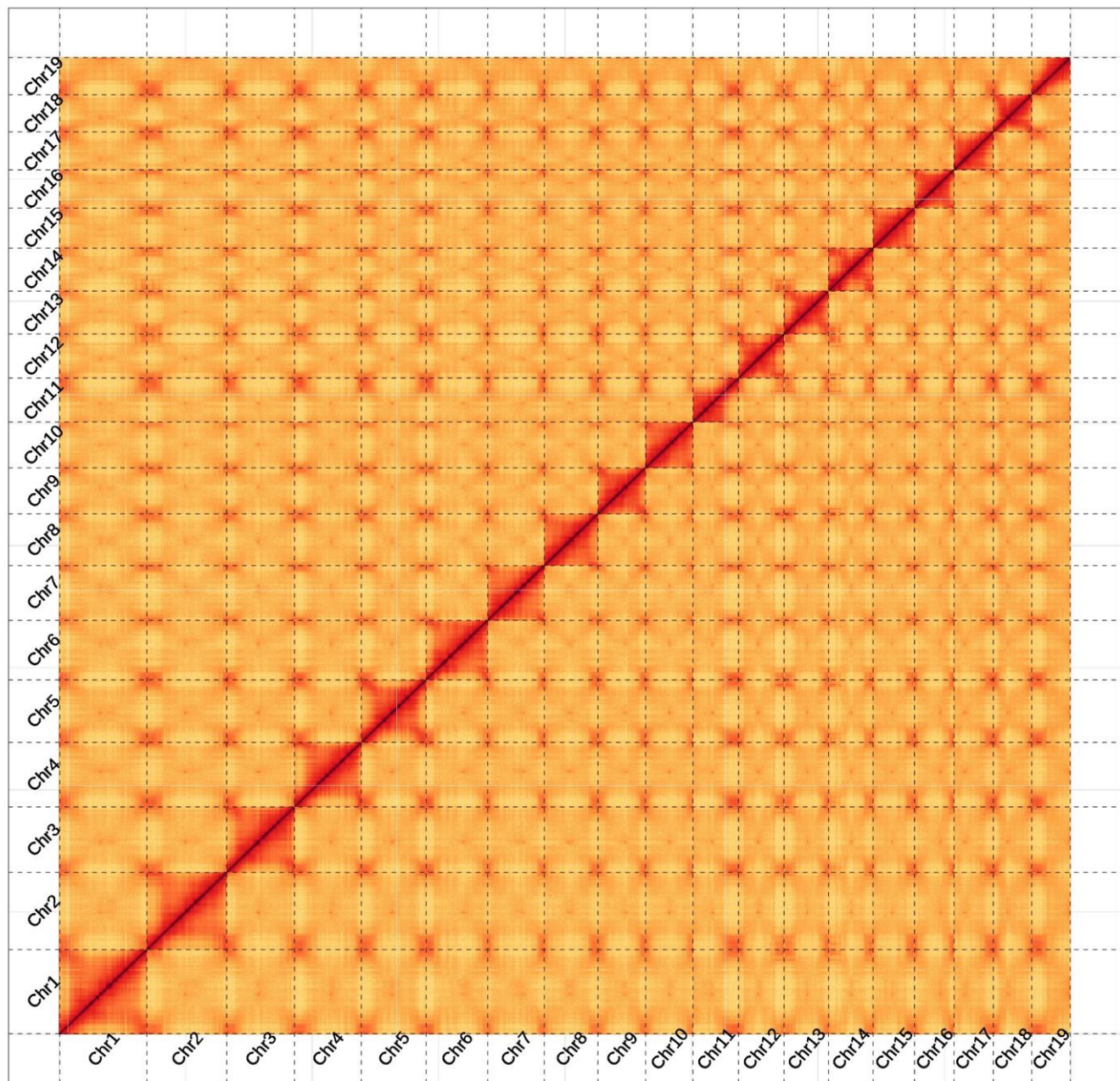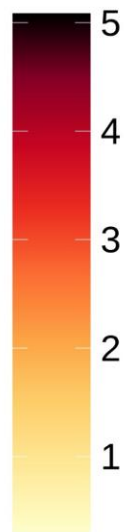

Supplement: Supplementary file 1 — Figure S1 [file 41597_2024_3617_MOESM1_ESM.pdf]
